# Bibliometric analysis of rehabilitation in Alzheimer’s disease (2000–2023): trends, hotspots and prospects

**DOI:** 10.3389/fnagi.2024.1457982

**Published:** 2024-12-03

**Authors:** Peng Jun, Hao Chengye, Wan Hui

**Affiliations:** ^1^Library and Information Center of Shanghai University Medicine & Health Sciences, Shanghai, China; ^2^Medical Management Office of Naval Medical University, Shanghai, China

**Keywords:** Alzheimer’s disease, rehabilitation, bibliometrics, CiteSpace, VOSviewer

## Abstract

**Background:**

Alzheimer’s disease (AD) is a complex neurodegenerative disease that leads to insidious deterioration of brain functions and is considered the sixth leading cause of death in the world. Multiple studies have shown that rehabilitation therapy is becoming an important field of AD research in recent years.

**Objective:**

We opted for bibliometric analysis to comprehensively summarize the advancements in the study of AD rehabilitation treatment, aiming to provide researchers with current trends and future research directions.

**Methods:**

All articles and reviews pertaining to rehabilitation treatment in Alzheimer’s disease from 2000 to 2023 were downloaded through Web of Science Core Collection. The results were subjected to bibliometric analysis using Microsoft Excel (2019 version), CiteSpace (6.3 R1 Advanced) and VOSviewer 1.6.20.

**Results:**

Overall, 1,284 publications were included. The number of publications was increasing yearly. The United States has published the most publications. University of Toronto has published the most papers of all institutions. NEUROPSYCHOLOGICAL REHABILITATION and ARCHIVES OF PHYSICAL MEDICINE AND REHABILITATION were the journals with the most studies and the most commonly cited, respectively. Clare L is the author with the highest productivity and co-citation. After analysis, the most common keywords are mild cognitive impairment, cognitive, impairment memory and executive function indicates that cognitive impairment is the main focus of research. Transcranial magnetic stimulation, cognitive rehabilitation, and physical activity/exercise are the hotspots of research at the present stage and are likely to continue.

**Conclusion:**

Distinguishing non pharmacological treatments at different stages of development is a research hotspot in AD rehabilitation; Sports intervention, brain functional imaging techniques represented by brain functional connectivity, virtual reality, and quality of life are research directions that need attention.

## 1 Introduction

Alzheimer’s disease (AD) is a complex neurodegenerative disease that leads to insidious deterioration of brain functions and is considered the sixth leading cause of death in the world (Ramachandran et al., 2021). There is no proven way to prevent Alzheimer’s disease, and there is currently no cure. However, because of the large number of people living with Alzheimer’s and other dementias worldwide (more than 55 million) and the devastating effect of dementia on individuals, families, communities and health care systems, finding ways to prevent, slow, better manage and cure Alzheimer’s and other dementias is a top priority for research centers around the globe (Alzheimer's disease facts and figures, 2024). Multiple studies have shown that rehabilitation therapy is becoming an important field of AD research in recent years. Bibliometric methods or “analysis” are now firmly established as scientific specialties and are an integral part of research evaluation methodology especially within the scientific and applied fields (Ellegaard and Wallin, 2015). This study comprehensively analyzes the relevant literature on AD rehabilitation treatment through bibliometric methods, aiming to obtain more quantitative information on research hotspots, development trends, cooperation between organizations, and the impact of publications, in order to enhance understanding of the current situation and development trends of AD rehabilitation and provide reference for later research.

## 2 Method

### 2.1 Data collection

**Figure 1:**
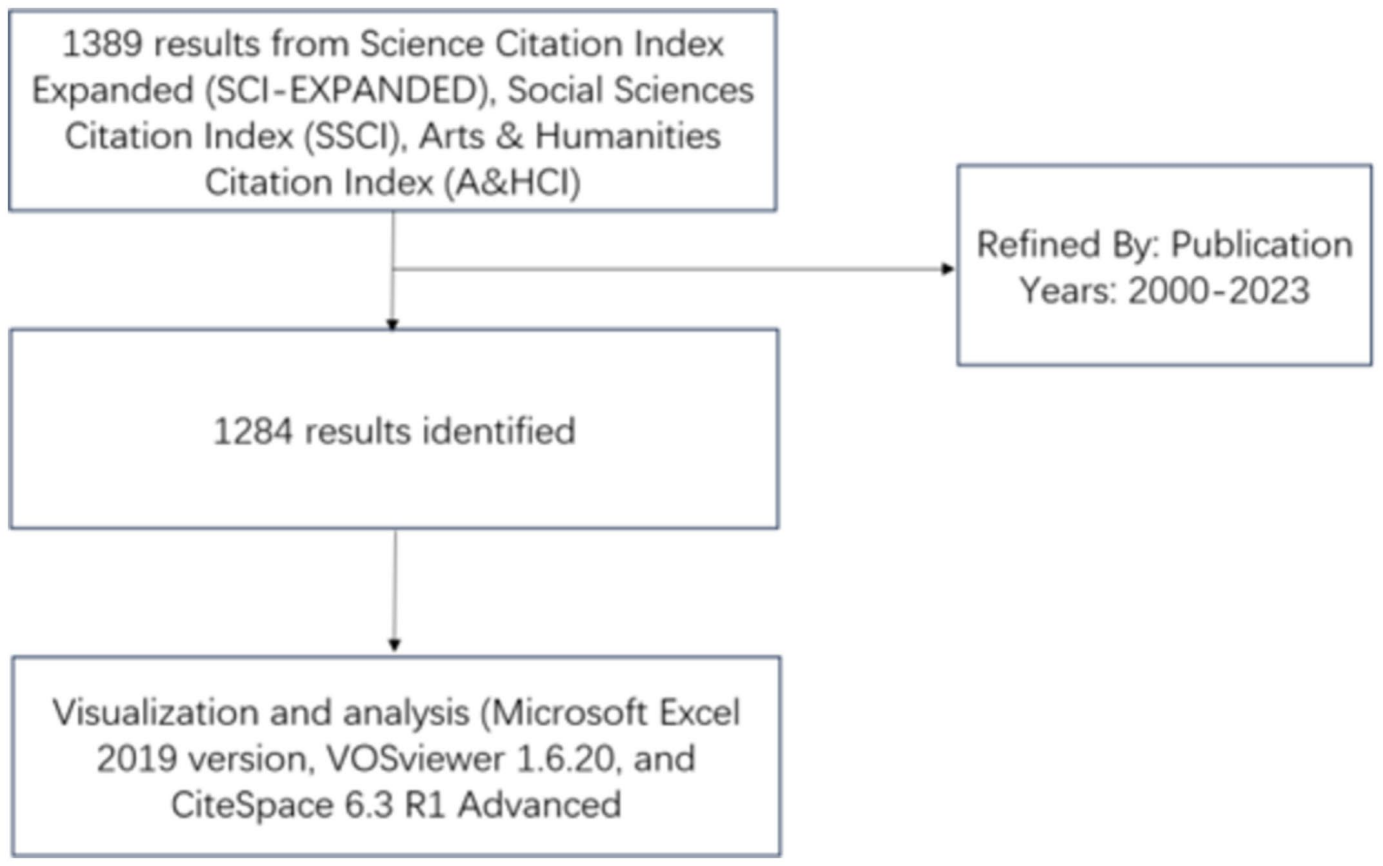
PRISMA flow diagram for AD rehabilitation research.

The Web of Science Core Collection (WoSCC) is the primary source database for data retrieval. As one of the largest and most comprehensive online databases in the world, WoSCC provides a wealth of highly authoritative and informative scientific research and analysis (Qin et al., 2022). We searched the Web of Science core collection database and analyzed the results using software such as Microsoft Excel (2019 version) and CiteSpace (6.3 R1 Advanced). The search formula is set to: ((TS = (“Alzheimer* Dementia*” OR “Dementia*, Alzheimer*” OR “Alzheimer* Disease*” OR “Disease*, Alzheimer*” OR “Dementia*, Alzheimer* Type” OR “Alzheimer* Type Dementia*” OR “Alzheimer-Type Dementia*” OR “Alzheimer* Type Dementia*” OR “Dementia*, Alzheimer-Type” OR “Alzheimer* Type Senile Dementia*” OR “Senile Dementia, Alzheimer Type” OR “Alzheimer* Sclerosis” OR “Sclerosis, Alzheimer*” OR “Alzheimer* Syndrome” OR “Syndrome, Alzheimer*”))) AND ((TS = (“rehabilita*” OR” Physiatr*” OR “physical medicine” OR “physical and rehabilitation medicine”))). Search for articles and reviews published in English between January 1, 2000 and December 31, 2023. Only Article and Review Article were included, and other types of articles such as Proceeding Paper, Editorial Material, Book Chapters, Early Access, Book Review, Meeting Abstract, Retracted Publication were excluded. The retrieval strategy identifies articles that involve the aforementioned search terms in their titles, abstracts, and keywords. A total of 1,284 articles were retrieved, all of which can be recognized by CiteSpace. In order to avoid data errors caused by subsequent database updates, all searches and downloads are completed within one day on March 1st, 2024. All articles are exported in TXT format (Figure 1).

### 2.2 Data analysis

After verifying the exported data, the two authors (PENG J and HAO C) deleted irrelevant articles and corrected spelling errors. Subsequently, the sorted dataset was imported into RStudio (2023 402), VOSviewer (1.6.20), and CiteSpace (6.3 R1 Advanced) for bibliometric analysis. Although VOSviewer and CiteSpace are mainly responsible for visualizing and analyzing data, the publication of literature over the years, distribution of country/region publication volume, and cooperation situation are drawn using R language’s ggplot, map, and other packages.

VOSviewer is a powerful bibliometric analysis software that helps with data extraction and processing (van Eck and Waltman, 2010). VOSviewer explores co-authorship, co-occurrence, citation, bibliographic coupling, and co-citation links in one of three possible representations: network, overlay, or density visualization (Arruda et al., 2022). In addition, CiteSpace is a Java-based application to analyze and visualize the hot spots and research frontiers in the scientific literature of a discipline or knowledge domain in a certain period with metrology, co-occurrence analysis and cluster analysis (Zhong et al., 2022). CiteSpace is a widely used bibliometric analysis software that provides an easy to understand understanding of research hotspots and evolutionary processes in specific fields, thus providing insights for future development directions (Synnestvedt et al., 2005). We used the software to transform quantitative literature data into visual maps and networks that provide key information including research hotspots, frontiers, and country cooperation between countries/regions/institutions. There are different nodes and links in various CiteSpace visual graphs. The nodes can represent different keywords, countries, institutions, journals, etc. The larger the node, the more emergence or citations in this area. The links between nodes represent their relationships. When countries/regions/institutions represented by node appear in the same document, there is a connecting line between the two. Different colors represent different years of issue. The newer the date, the warmer the color of the node/link, and the earlier the date, the cooler the color of the node/link. The centrality indicates the importance of this node in the network, and in CiteSpace, nodes with purple rings are considered to be centrally located with high centrality (Fu et al., 2023). High betweenness centrality values identify potentially revolutionary scientific publications as well as gatekeepers in social networks (Chen et al., 2010).

Our study also used RStudio, VOSviewer, and CiteSpace to analyze country/regional distribution, institutional distribution, author collaboration and distribution, keyword distribution and collaboration, double graph overlap of journals, and keyword explosion.

## 3 Results

### 3.1 Annual publication and citation volume

**Figure 2:**
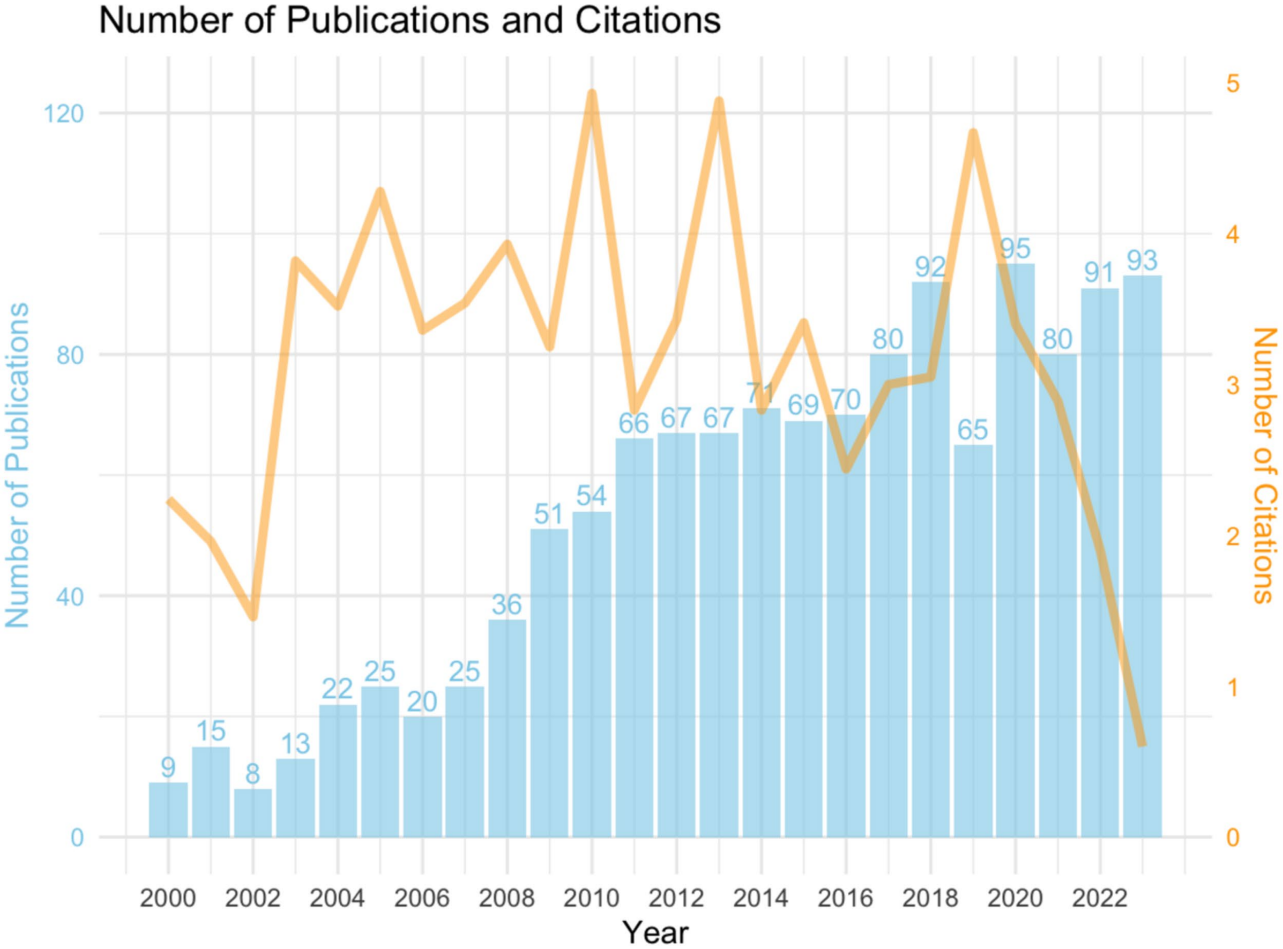
Annual publications on Alzheimer’s disease rehabilitation research.

From 2000 to 2023, a total of 1,284 papers were ultimately used for data analysis. Analyzing the annual publication volume (Figure 2), it was found that it is mainly divided into three stages.

The first stage (2000–2010): The overall number of publications showed an upward trend (278 articles), with an average annual publication volume of 25.27 articles and an average annual growth rate of 20%, indicating that AD rehabilitation therapy has attracted the attention of researchers and has developed rapidly.

Phase 2 (2011–2016): The number of publications entered the platform stage (410 articles), with an average annual publication volume of about 68.33 articles and an average annual growth rate of 1%, indicating that the research scale in this field is stabilizing and gradually entering a mature stage.

In the third stage (2017–2023), the number of publications showed a fluctuating upward trend again (596 articles), with an average annual increase of 85.14 articles and an average annual growth rate of 2%. Among them, the average annual increase in 2020, 2022, and 2023 was over 90 articles, indicating that new methods and technologies may provide researchers with new exploration directions. In 2019, the number of published papers dropped to 65, which may be related to the global COVID-19 outbreak and the suspension of some research work. Overall, rehabilitation therapy is showing a rapid growth trend in the field of AD research, and there is still value and potential for further exploration. During the COVID-19 pandemic, Alzheimer’s disease clinical trials faced multiple disruptions, including the suspension of in-person visits, reduced patient participation, and delays in treatment interventions, impacting data collection and research progress (Schneider et al., 2021).

**Figure 3:**
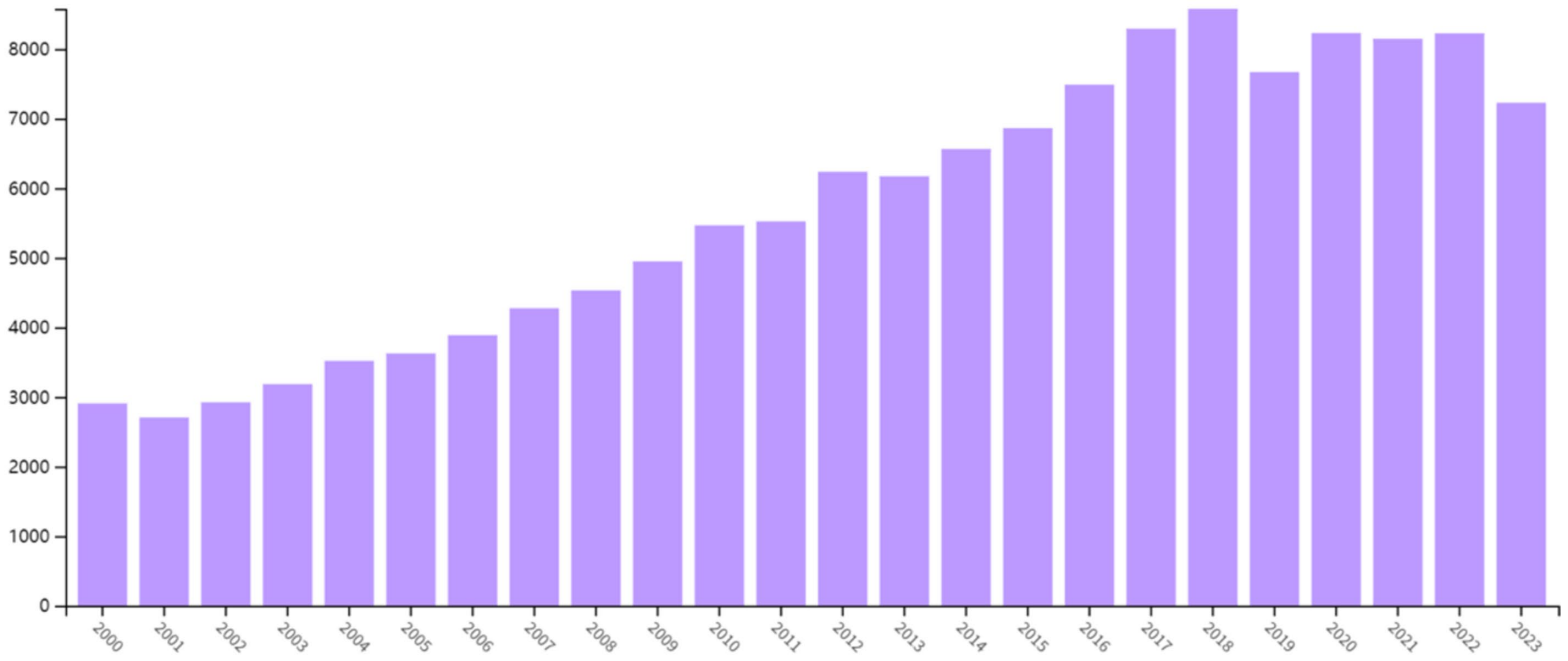
Annual publications on Alzheimer’s disease research.

We conducted a further analysis of the overall literature on Alzheimer’s disease (AD) and the literature on AD rehabilitation (Figure 3). Although the total number of AD-related publications increased from 2,906 in 2000 to 7,226 in 2023—an approximately 2.5-fold increase—the annual growth rate has remained moderate. From 2000 to 2016, the average annual growth rate was around 6%, indicating a relatively sustained and stable growth trend. Although there was a −6.95% decline in 2001, the growth rate recovered to around 8% in the following years. These fluctuations did not significantly affect the long-term trend, and overall growth remained steady. From 2017 to 2023, the growth in publication volume began to slow, with some years even showing negative growth. In 2023, the publication volume was 7,226, down −12.14% from 8,224 in 2022, indicating that the volume of publications in this phase has entered a period of saturation or slowed growth, lacking a significant expansion trend. Overall, the growth pattern of publication volume can be described as gradual and long-term stable, consistent with common patterns of large-scale literature development.

In contrast, although the number of publications in the field of AD rehabilitation has also increased, its growth is more pronounced compared to the overall AD literature. This comparison further supports the idea that AD rehabilitation literature is gaining increasing attention in the scientific community.

### 3.2 Distribution of countries and regions

**Figure 4:**
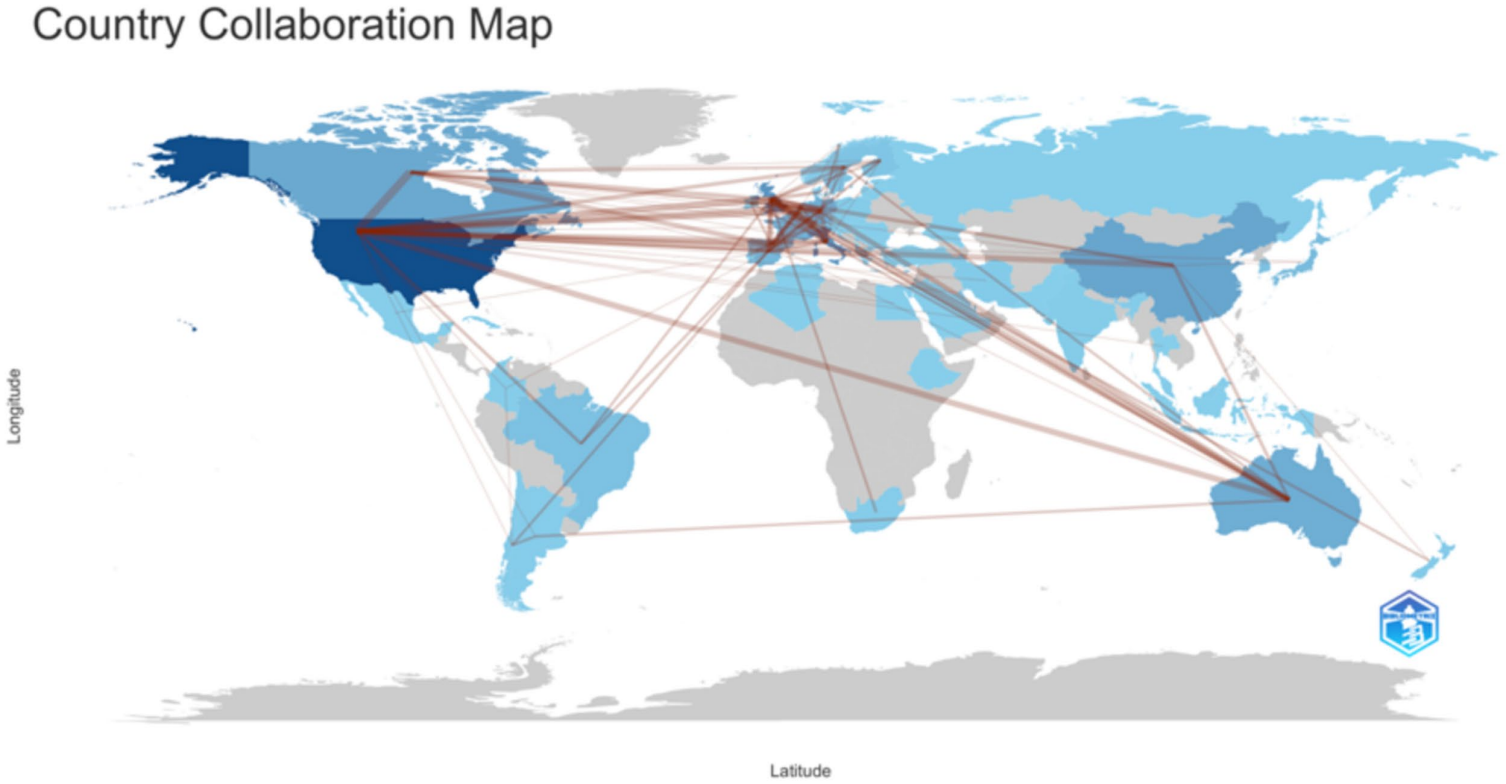
Distributions of countries/regions postings and collaboration network.

There are a total of 66 countries participating in the study. Taking into account the number of publications, citation frequency, and national cooperation networks, it is possible to gain a preliminary understanding of the status and role of different countries in academic output and scientific research cooperation in this field (Figure 4, Table 1). Based on the country of the corresponding author, calculate the number of publications in each country. The United States ranks first in terms of publication volume, citation frequency, and betweenness centrality; Next is Italy; The UK and China rank third in terms of publication volume (both with 96 articles), but the UK’s betweenness centrality (211.84) is significantly higher than China’s (14.62). These findings suggest several strategic opportunities for future international collaboration:

1. Strengthening connections between high-output Asian institutions and established Western research networks could enhance global knowledge exchange.
2. Supporting emerging research hubs in developing their international collaboration capabilities while maintaining existing strong networks.
3. Encouraging more active participation from underrepresented regions to achieve a more balanced global research network.

**Table 1:** Top 10 countries/regions in terms of volume of publications.

No.	Country	Publication volume	Percentage of publications	Citation frequency	Betweenness centrality
1	United States of America	307	23.92%	15,578	308.15
2	Italy	137	10.67%	3,898	53.43
3	United Kingdom	96	7.48%	4,164	211.84
4	China	96	7.48%	1,774	14.62
5	Canada	71	5.53%	2,463	55.91
6	Australia	69	5.37%	2,614	55.6
7	Germany	56	4.36%	1,107	76.5
8	France	54	4.21%	1,547	86.76
9	Spain	48	3.74%	1,246	42.1
10	Japan	43	3.35%	737	2.46

### 3.3 Institutional analysis

A total of 1,664 institutions have published research on AD rehabilitation treatment (Table 2). In terms of the number of publications, the top three institutions are the University of Toronto, the University of California system, and the United States Veterans Health Administration. However, in terms of betweenness centrality, the University of California system (185.14), Johns Hopkins University (148.75), and University of London (109.44) have made greater contributions to promoting cross institutional collaborative research compared to the University of Toronto (30.26).

**Table 2:** Top 10 institutions in terms of volume of publications.

No.	Institution	Publication volume	Percentage of publications	betweenness centrality
1	University of Toronto	77	6.00%	30.26
2	University of California System	58	4.52%	185.14
3	Veterans Health Administration	52	4.05%	74.82
4	University of London	46	3.58%	109.44
5	University of Helsinki	41	3.19%	27.29
6	Paris Public Hospitals Group	40	3.12%	29.02
7	University College London	40	3.12%	70.74
8	Sorbonne University	37	2.88%	27.92
9	Johns Hopkins University	36	2.80%	148.75
10	University of São Paulo	35	2.73%	0

Based on the above analysis, research institutions in AD rehabilitation studies can be categorized into two types: high-output oriented institutions represented by the University of Toronto, which demonstrate strong research productivity but moderate network centrality, often with advantages in specific research directions and clinical data accumulation.

The other type is network hub institutions represented by the University of California System and Johns Hopkins University, which, despite relatively fewer publications, play crucial connecting roles in research networks through their higher betweenness centrality.

This institutional distribution pattern suggests potential benefits in encouraging collaboration between these complementary types of institutions, such as through joint research programs or shared data platforms, to promote the integration and optimal utilization of research resources.

### 3.4 Magazine contributions

1,284 articles were published in 462 journals, and bibliometric analysis was conducted on these journals, covering multiple academic influence indicators such as publication volume, citation frequency, and H-index.

In terms of publication volume, NEUROPSYCHOLOGICAL REHABILITATION has the highest publication volume, but its impact factor and JCR partition are relatively low; ARCHIVES OF PHYSICAL MEDICINE AND REHABILITATION ranks second in terms of publication volume, but has the highest citation frequency, indicating that the journal has higher academic influence and authority. The third and fourth place in terms of publication volume are in some professional journals that have shown outstanding performance in specific research directions, such as JOURNAL OF ALZHEIMERS DISEASE, which has the highest publication volume in the field of Alzheimer’s disease research (24 articles, IF = 4), and FRONTIERS IN AGING NEUROSCIENCE, which focuses on research in geriatric neuroscience and neurodegenerative diseases (IF = 4.8, 22 articles), reflecting the importance of specialized and distinctive development paths (Table 3).

**Table 3:** Top ten journals in terms of number of publications.

No.	Journal	Publication volume	H-index	Citation frequency	JIF2022	JCR
1	NEUROPSYCHOLOGICAL REHABILITATION	51	27	1,395	2.7	Q3
2	ARCHIVES OF PHYSICAL MEDICINE AND REHABILITATION	35	19	2,157	4.3	Q1
3	JOURNAL OF ALZHEIMERS DISEASE	24	11	397	4	Q2
4	FRONTIERS IN AGING NEUROSCIENCE	22	13	589	4.8	Q2
5	AGING & MENTAL HEALTH	21	12	635	3.4	Q2
6	BMC GERIATRICS	20	9	435	4.1	Q1
7	ARCHIVES OF GERONTOLOGY AND GERIATRICS	18	10	406	4	Q2
8	INTERNATIONAL JOURNAL OF GERIATRIC PSYCHIATRY	17	14	839	4	Q2
9	PLOS ONE	17	12	629	3.7	Q2
10	TOPICS IN GERIATRIC REHABILITATION	17	8	148	0.5	Q4

In terms of influencing factors, Table 4 summarizes 10 top journals located in the Q1 interval. Among them, LANCET, JAMA-JOURNAL OF THE AMERICAN MEDICAL ASSOCIATION, and LANCET have only published 1–2 papers in this field, but their IF ranks in the top three, corresponding to the titles of published papers: Senile fatigue; A randomized controlled trial of exercise and behavioral management in Alzheimer’s disease patients; Methods for repairing dementia patients; Elderly people with a history of traumatic brain injury have decreased cognitive abilities; Neuropsychological and clinical heterogeneity of cognitive impairment and dementia in Parkinson’s disease patients (Table 4).

**Table 4:** The top 10 journals with the highest impact factors.

No.	Journal	Publication volume	H-index	Citation frequency	JIF2022	JCR
1	LANCET	1	1	817	168.9	Q1
2	JAMA-JOURNAL OF THE AMERICAN MEDICAL ASSOCIATION	2	2	518	120.7	Q1
3	LANCET NEUROLOGY	2	2	808	48	Q1
4	JAMA INTERNAL MEDICINE	2	2	1,217	39	Q1
5	NATURE REVIEWS NEUROLOGY	2	2	150	38.1	Q1
6	JAMA NEUROLOGY	2	2	160	29	Q1
7	CIRCULATION RESEARCH	1	1	139	20.1	Q1
8	ACS NANO	1	1	9	17.1	Q1
9	AMERICAN PSYCHOLOGIST	1	1	38	16.4	Q1
10	IEEE JOURNAL ON SELECTED AREAS IN COMMUNICATIONS	1	1	9	16.4	Q1

### 3.5 Knowledge graph of double image overlay in journals

**Figure 5:**
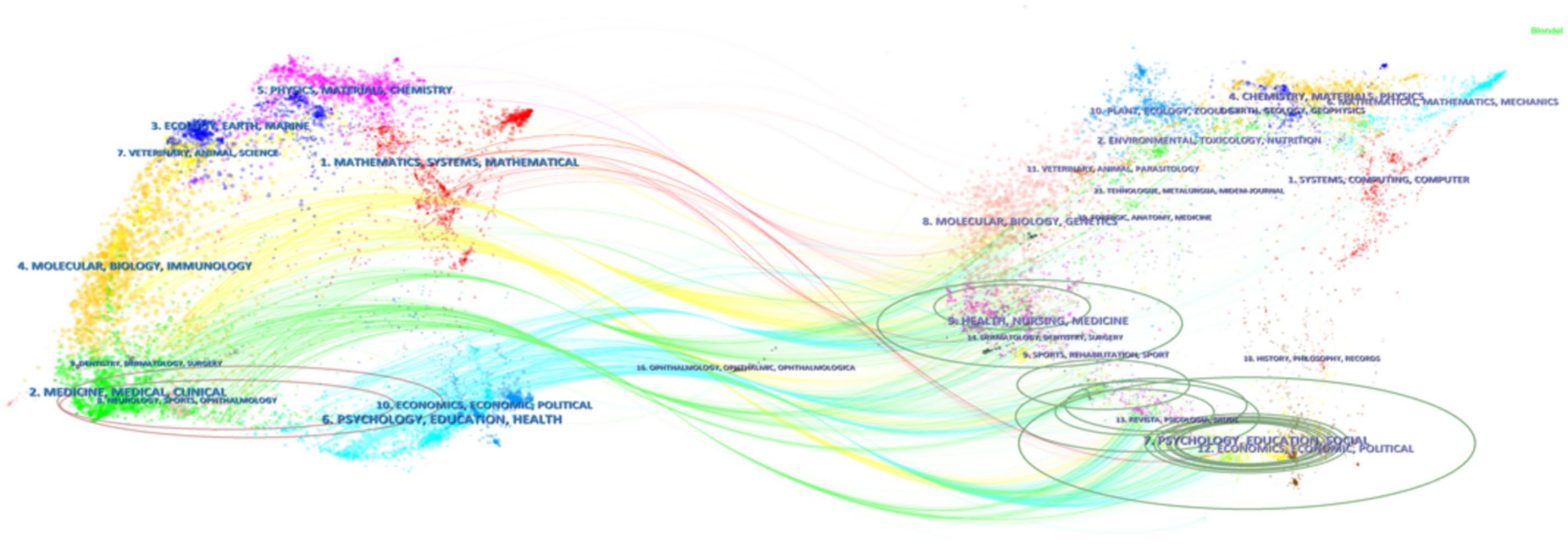
Journal biplot overlay mapping.

Use CiteSpace software to draw a knowledge graph of journal Dual map Overlay (Figure 5) to reveal the disciplinary distribution and cross fusion characteristics of journals in this research field. From the overlapping results of the two graphs, it can be seen that the cited journals are mainly distributed in Zone 2 (medical, clinical) and Zone 6 (psychology) on the left side of the graph, (education, health); The cited journals are mainly distributed in areas 7 (psychology, education, social), 8 (health, nursing, medicine), and 9 (sports) on the right side of the figure, (rehabilitation, sport). Overall, AD rehabilitation research has characteristics that span both natural and social sciences. Psychology, education, medicine, and health are not only the citation basis of research, but also the application fields of research results.

### 3.6 Author collaboration and co citation network analysis

**Figure 6:**
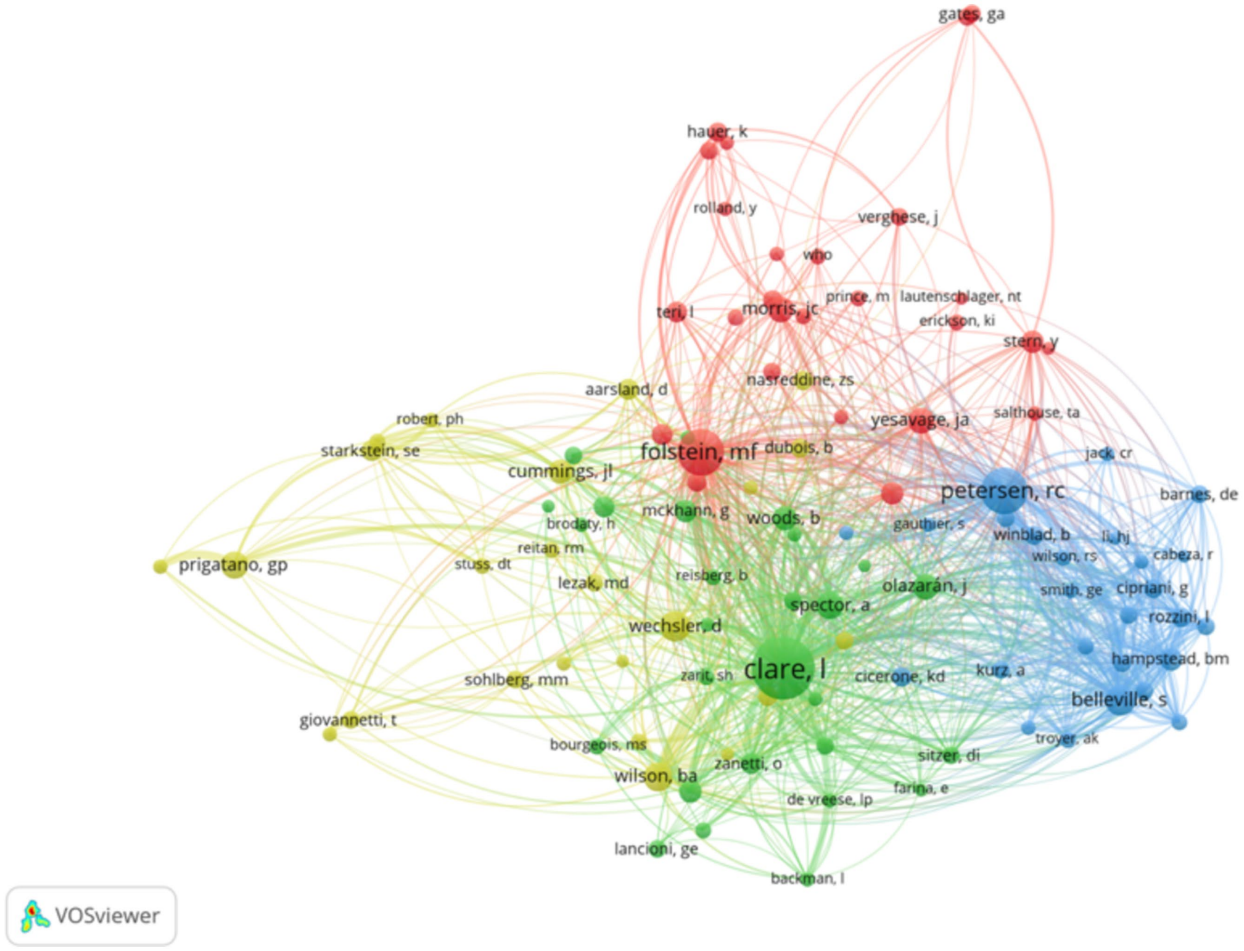
Authors’ co-citation network.

5,779 authors have completed 1,286 research papers on AD rehabilitation. By analyzing the author’s publication volume, collaborative network, and co citation network, we can evaluate the core authors and their academic influence in this field from multiple dimensions (Figure 6).

Based on various indicators, scholars such as Clare L., Hampstead B.M., and Belleville S. have shown outstanding performance. Clare L. ranks first in terms of publication volume and citation frequency with 20 papers and 1,544 citations, and its H-index is also as high as 15, reflecting a stable and sustained high-level academic output. Although Hampstead B.M. has a relatively small number of papers (12 papers), the frequency of single citations is high, with a total of 454 citations and an H-index of 9, indicating the high impact of their papers.

From the perspective of scholar co citation networks, some important authors have high betweenness centrality, such as Petersen R.C. (287.6), Belleville S. (137.1), Winblad B. (54.2), Sitzer D.I. (56.0), etc., indicating that their research has played a key role in connecting different disciplinary teams in the field and promoting cross team cooperation. In contrast, the mediating neutrality of Clare L. (32.2) and Hampstead B.M. (14.9) is relatively low, which may be related to their more focused and consistent research direction.

It is related to the relative aggregation of networks. In addition, some scholars, although producing a small number of papers, stand out due to the high influence of individual papers, such as Yaffe K. Although there are only four papers, the citation frequency of a single paper is as high as 1,272 times; Gates G.A.’s four papers were cited a total of 1,137 times, while Lin F.R.’s two papers were cited 1,054 times, both of which demonstrate important academic contributions.

### 3.7 Reference cluster analysis

**Figure 7:**
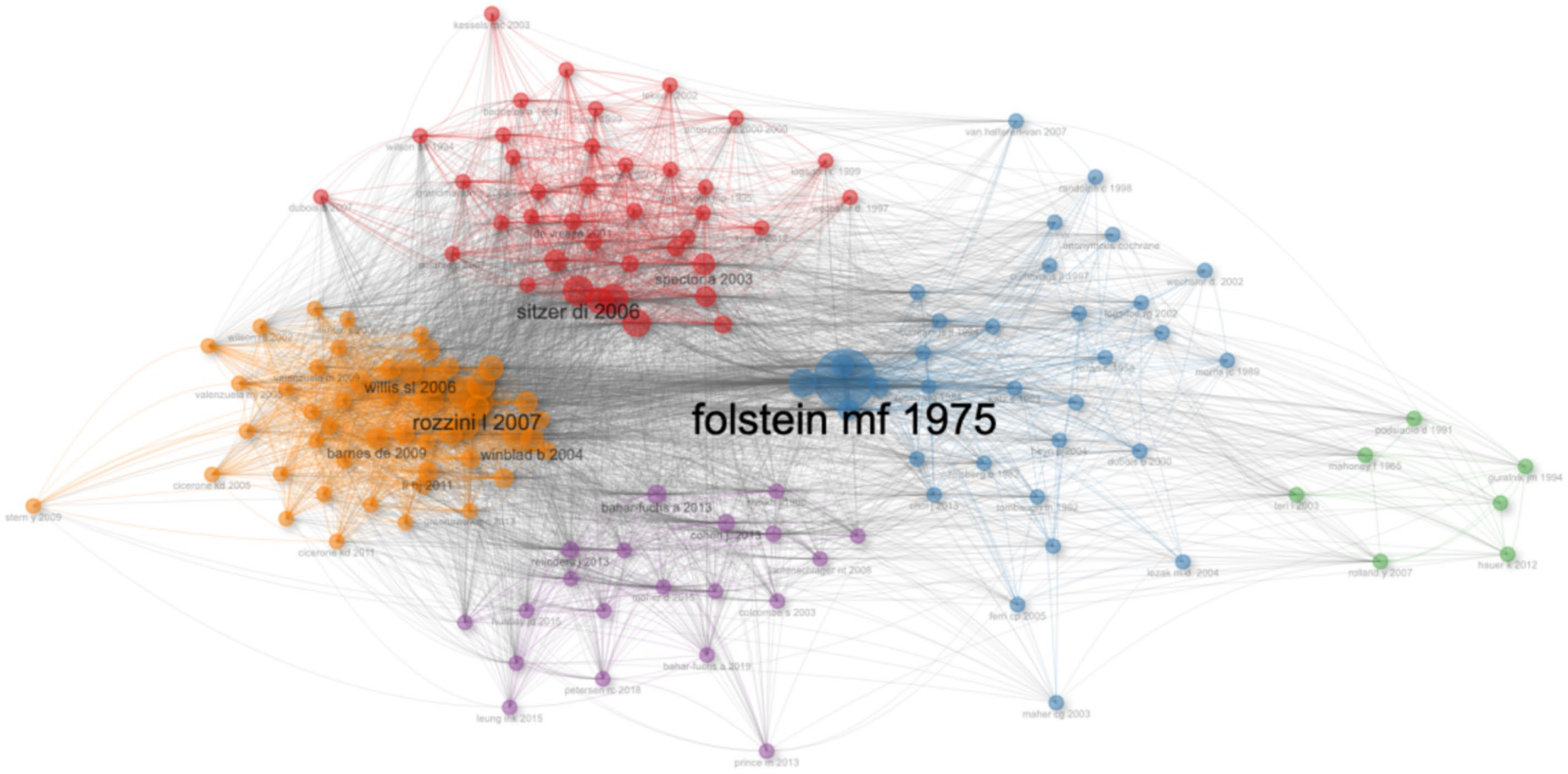
Reference cluster analysis mapping.

Perform cluster analysis on the cited references. Overall, these clusters mainly focus on the diagnosis, evaluation, screening, and intervention of AD, with a focus on methodological research, and have strong universality and citation significance. For example, in Cluster 1, Folstein MF (1975) has the highest centrality, and its developed Mini Mental State Examination (MMSE) holds an important position in the diagnosis of AD (Folstein et al., 1975). Other key authors include McKhann G (1984, 2011) and Yesavage JA (1983), whose work involves diagnostic criteria and cognitive assessment scales for AD (McKhann et al., 1984; Yesavage, 1983; McKhann et al., 2011). The main authors of Cluster 2 include Clare L (2004, 2003, 2002), Sitzer DI (2006), and Olazarán J (2010, 2004), whose research mainly focuses on cognitive rehabilitation, memory intervention, and treatment guidelines (Clare, 2004; Clare et al., 2003; Clare et al., 2002; Sitzer et al., 2006; Olazaran et al., 2010; Olazaran et al., 2004). The study of Mahoney F (1965) in Cluster 3 has a significant impact on functional assessment (Mahoney and Barthel, 1965), while Rolland Y (2007) and Teri L (2003) et al. focused on physical activity and behavioral interventions (Rolland et al., 2007; Teri et al., 2003). The Timed Up and Go test developed by Podsiadlo D (1991) is also an important tool for functional assessment (Podsiadlo and Richardson, 1991) (Figure 7).

### 3.8 Highly cited references

Table 5 lists the top 10 papers with the most citations on AD rehabilitation therapy. Among them, the paper with the most citations (338 times) is “Mini mental state”: a practical method for grading the cognitive state of patients for the clinician, The Mini Mental State Examination (MMSE) developed by author Folstein et al. provides a simple, fast, and effective tool for early screening of dementia, and lays the foundation for the subsequent development of dementia diagnostic criteria (Folstein et al., 1975). The second ranked paper with 103 citations is “Clinical diagnosis of Alzheimer’s disease: Report of the NINCDS-ADRDA Work Group under the organs of Department of Health and Human Services Task Force on Alzheimer’s Disease.” The authors, McKann et al., standardized the diagnostic criteria and process for Alzheimer’s disease, which comprehensively consider medical history, physical examination, cognitive function assessment, laboratory tests, and imaging evidence, making the diagnosis of AD more objective and accurate. It also provides a basis for distinguishing AD from other types of dementia (McKhann et al., 1984). The third ranked paper is “Cognitive training and cognitive rehabilitation for people with early stage Alzheimer’s disease: A review.” The author Clare focuses on the field of neuropsychological rehabilitation, exploring how to help dementia patients maintain and improve their quality of life through various means such as cognitive training, daily life activity training, and psychological support (Clare et al., 2003). This study emphasizes a patient-centered approach, focusing on the subjective feelings and actual benefits of patients, and opens up a new path for non pharmacological treatment of AD. Overall, these highly cited references mainly focus on early screening tools, diagnostic criteria, neuropsychological rehabilitation, cognitive training, and cognitive rehabilitation for AD, laying a solid theoretical foundation for subsequent research.

**Table 5:** The top 10 cited references in terms of frequency.

Title	Author	Year	Number of Citations
“Mini-mental state”: A practical method for grading the cognitive state of patients for the clinician	FOLSTEIN MF	1975	338
Clinical diagnosis of Alzheimer’s disease: Report of the NINCDS_ADRDA Work Group under the auspices of Department of Health and Human Services Task Force on Alzheimer’s Disease	MCKHANN G	1984	103
Cognitive training and cognitive rehabilitation for people with early-stage Alzheimer’s disease: A review	CLARE L	2004	86
Mild cognitive impairment: clinical characterization and outcome	PETERSEN RC	1999	80
Mild cognitive impairment as a diagnostic entity	PETERSEN RC	2004	78
Development and validation of a geriatric depression screening scale: a preliminary report	YESAVAGE JA	1982	76
Cognitive rehabilitation and cognitive training for early-stage Alzheimer’s disease and vascular dementia	CLARE L	2003	72
The Montreal Cognitive Assessment, MoCA: A Brief Screening Tool For Mild Cognitive Impairment	NASREDDINE ZS	2005	72
Cognitive training in Alzheimer’s disease: a meta-analysis of the literature	SITZER DI	2006	68
Effectiveness of a cognitive rehabilitation program in mild dementia (MD) and mild cognitive impairment (MCI): A case control study	TALASSI E	2007	67

We found that these highly cited papers not only laid the foundation for Alzheimer’s disease research but also advanced the field of cognitive rehabilitation. From the MMSE developed by Folstein et al. (1975). to the MoCA by Nasreddine et al. (2005), we observe the evolution of assessment tools. The work of McKhann et al. (1984), Petersen et al. (1999) and Petersen (2004) provided standardized diagnostic criteria for AD and MCI respectively, making early identification and intervention possible. Studies by Clare et al. (2003), Sitzer et al. (2006) and Clare and Woods (2004), delved into the effects of cognitive training and rehabilitation, providing empirical support for non-pharmacological treatment methods.

These studies collectively reveal the potential benefits of cognitive rehabilitation for Alzheimer’s disease patients, especially in the early stages of the disease. However, they also expose some limitations in current research, such as small sample sizes and short follow-up periods. Future research directions may include developing more precise early diagnostic tools, designing long-term follow-up studies, exploring personalized cognitive rehabilitation programs, and examining the synergistic effects of cognitive rehabilitation and pharmacological treatments.

### 3.9 Reference emergence analysis

**Figure 8:**
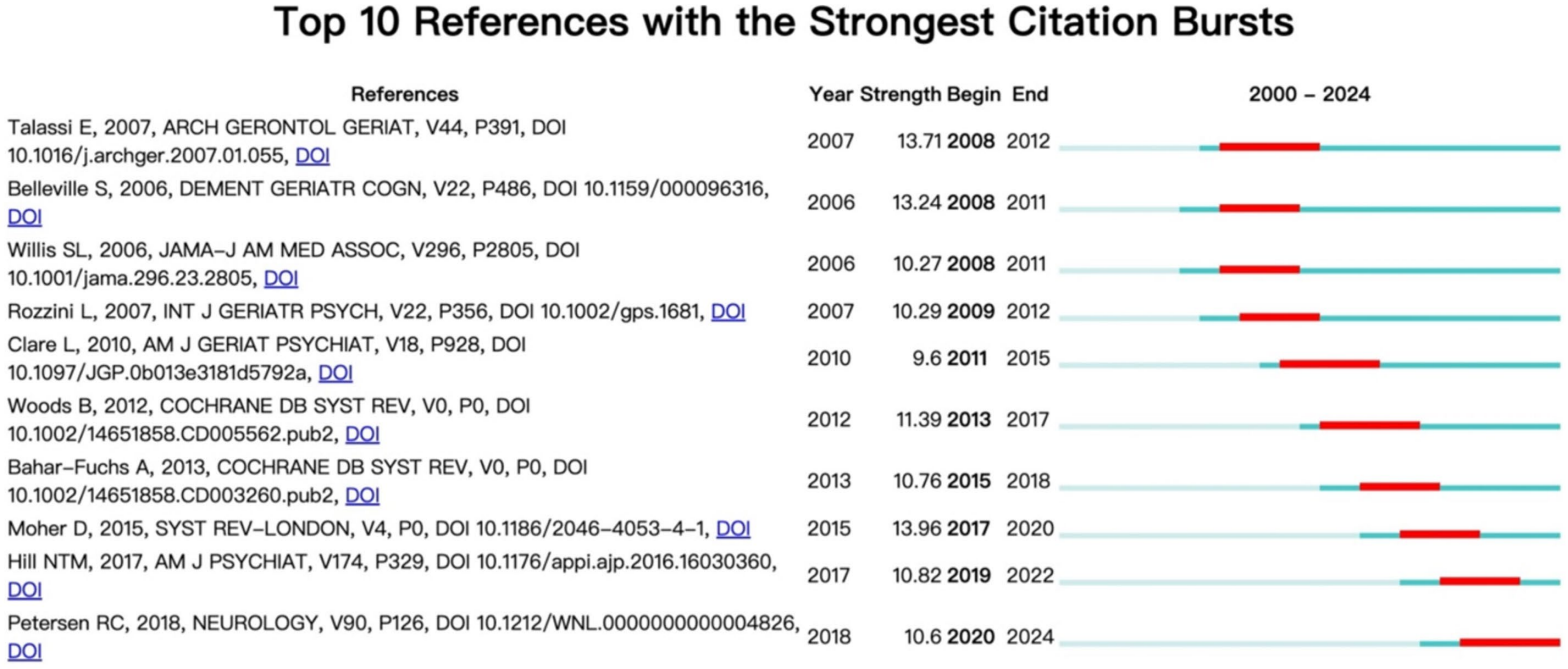
Top 10 reference with the strongest citation bursts.

According to the analysis of citation emergence data, the phased characteristics and trends of AD rehabilitation research hotspots can be seen (Figure 8). This chart shows the top 25 keywords with the strongest citation bursts in Alzheimer’s disease and rehabilitation research from 2000 to 2024. Each keyword’s “burst period” (red bar) represents a phase (from “Begin” to “End”) when it saw a rapid increase in citations, indicating heightened research interest. The higher the burst strength (Strength), the greater the keyword’s impact during that time. The green–blue bar shows the keyword’s presence outside the burst period, indicating a steadier citation rate.

Early research (2008–2012): Focusing primarily on cognitive training and rehabilitation efficacy in patients with mild cognitive impairment (MCI) and early AD. Talasi et al. adopted a computerized cognitive training program and found that systematic cognitive rehabilitation training can improve the cognitive and emotional states of MCI and mild AD patients (Talassi et al., 2007); Belleville et al. and Willis et al. also support the positive effects of cognitive training on memory and daily function in MCI patients (Belleville et al., 2006); Rozzini et al. explored the therapeutic effect of cognitive rehabilitation training combined with drug therapy (cholinesterase inhibitors) on MCI and found that combination therapy was superior to drug therapy alone in improving cognition, emotion, and other aspects (Rozzini et al., 2007). In short, this stage of research has confirmed the positive effects of cognitive training and rehabilitation as non pharmacological treatment methods in intervening and delaying the progression of Alzheimer’s disease, and has begun to focus on the comprehensive efficacy of drug combined non pharmacological treatment.

In the mid-term stage (2011–2015), randomized controlled trials and systematic reviews have received much attention. Clare et al. designed a personalized, specific functional goal oriented cognitive rehabilitation training and found that this targeted cognitive rehabilitation program significantly improved subjective evaluation indicators such as goal achievement and satisfaction, as well as objective cognitive behavior performance in early AD patients (Clare et al., 2010). Woods et al.’s systematic review summarized and analyzed evidence from multiple randomized controlled trials, and found that implementing cognitive stimulation regimens in mild to moderate AD patients can further improve cognitive function in addition to drug therapy, and have a positive impact on quality of life and happiness (Woods et al., 2012). It can be seen that during this stage, high-quality evidence-based medicine evidence for AD cognitive rehabilitation training has emerged, and empirical articles on systematic reviews and meta-analysis have received more attention and citations. Meanwhile, personalized and targeted cognitive rehabilitation programs for specific functional impairments have become a new research hotspot.

Recent stage (2017–2022): Guidelines have become a new high citation field. Hill et al.’s meta-analysis included 17 MCI and 11 AD randomized controlled trials, and found that computerized cognitive training had a moderate improvement effect on overall cognition, attention, working memory, learning memory (excluding verbal memory), and social psychological function (including depression) in MCI patients, but the effect on AD patients was relatively weak (Hill et al., 2017); In the updated AAN guidelines, Petersen et al. recommend non pharmacological measures such as assessing MCI risk, monitoring cognitive function changes, and suggesting regular exercise based on systematic evaluation evidence, and allow for careful consideration of drug therapy use. However, patients should be informed that there is currently no conclusive evidence of efficacy. This guideline embodies the concept of evidence-based medicine and has important value in guiding clinical practice. This stage of research suggests that computerized cognitive training, non pharmacological measures, and medication therapy may benefit MCI patients, but their effects on AD patients are relatively limited.

## 4 Hotspot frontier analysis

### 4.1 Keyword frequency and clustering analysis

By extracting and statistically analyzing high-frequency keywords from literature, it can be seen that there are hotspots and cutting-edge directions in this research field (Table 6). Excluding high-frequency search terms such as “Alzheimer’s disease, rehabilitation, dementia, and the elderly,” the frequency of words such as mild cognitive impairment (MCI, 128 times) and cognitive impairment (cognitive impairment, 61 times) is higher. This indicates that research on AD rehabilitation should be conducted according to different stages. The high prevalence of memory (127 times) and executive function (33 times) indicates that cognitive impairment is the main focus of research. Transcranial magnetic stimulation (21 times), cognitive rehabilitation (47 times), and physical activity/exercise (86 times) have become high-frequency terms, indicating that non pharmacological treatment methods are increasingly attracting the attention of researchers.

**Table 6:** Keyword frequency co-occurrence mapping.

Words	Occurrences
Alzheimers-disease	1,043
rehabilitation	310
dementia	291
older-adults	240
people	176
impairment	154
memory	127
quality-of-life	103
performance	102
mild cognitive impairment	100
intervention	95
risk	72
depression	70
randomized controlled-trial	70
program	66
efficacy	62
cognitive impairment	61
deficits	61
Parkinsons-disease	58
individuals	57

**Figure 9:**
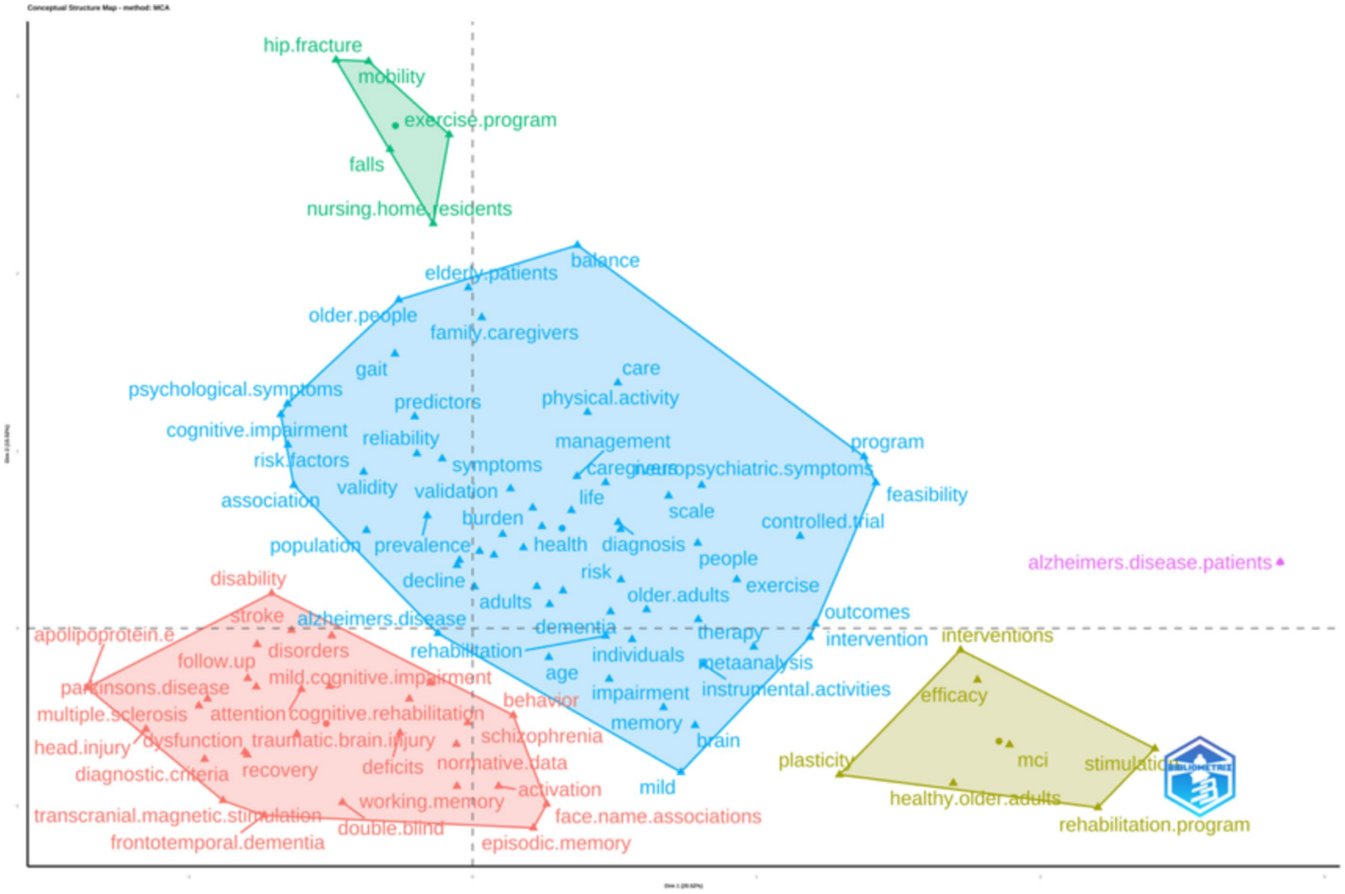
Cluster analysis of the top 100 keywords in terms of word frequency.

The clustering results generated through Multiple Correspondence Analysis (MCA) (Figure 9) illustrate the relationships among different keywords in Alzheimer’s disease and related rehabilitation research. Each colored area represents a distinct thematic cluster, with keywords positioned closer together indicating stronger associations. The analysis reveals that the research primarily focuses on the following four themes:

The first type (Cluster 1) focuses on the basic disease characteristics of AD. High frequency words include symptoms, diagnosis, treatment, population validity, risk factors, care, quality of life, etc., mainly describing the overall situation of AD.

Cluster 2 focuses on the differential diagnosis of different types of dementia. High frequency words include head injury, traumatic brain injury, stroke, Parkinson’s disease, multiple sclerosis, frontotemporal dementia, mild cognitive impairment, dysfunction, schizophrenia, etc. The main focus is to compare the diagnostic criteria and neurological and psychiatric symptoms of different types of dementia syndromes.

The third category (Cluster 3) focuses on intervention methods and rehabilitation measures for AD treatment. High frequency words include efficacy, interventions, rehabilitation programs, transcranial magnetic stimulation, plasticity, etc. By developing a systematic cognitive or physical rehabilitation training plan and utilizing non-invasive brain stimulation, they promote neural cell remodeling and achieve the goal of improving patient symptoms.

The fourth category (Cluster 4) focuses on common issues that affect self-care abilities in the elderly population. High frequency words include balance, mobility, falls, hip fractures, exercise programs, and nursing home residents. Among them, falls, fractures, etc. are important causes of disability and disability in elderly people. Walking, exercise, and other issues reflect the independent living ability of elderly patients, and also involve health management issues for the elderly population in nursing homes.

### 4.2 Keyword emergence analysis

**Figure 10:**
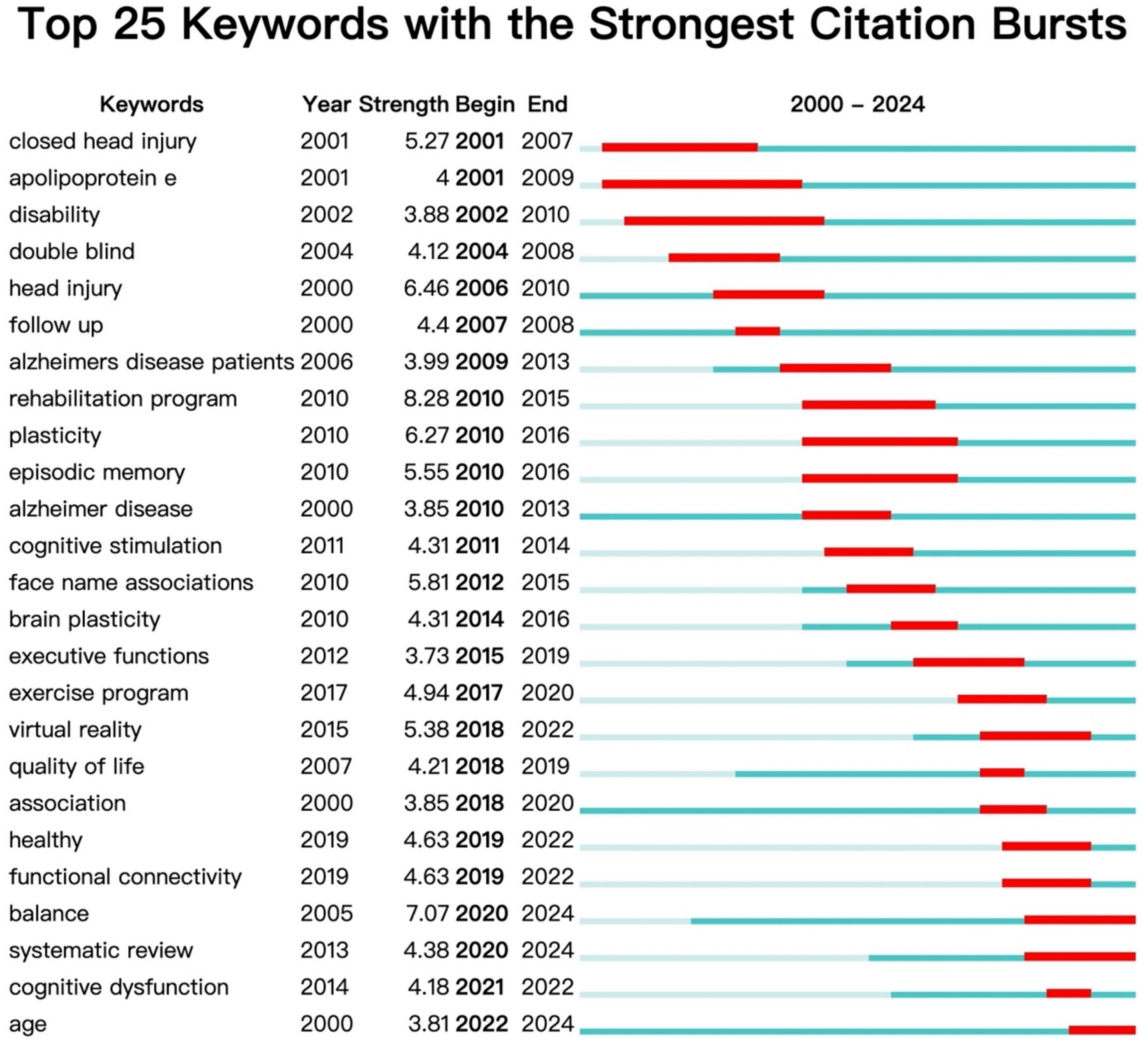
Top 25 keywords with the strongest citation bursts.

According to CiteSpace’s keyword emergence analysis (Figure 10), it can be seen that the trend of hot topic evolution in this research field is:

In the early stages (2001–2010), keywords such as closed head injury, apolipoprotein E, and head injury emerged, indicating that research at that time mainly focused on traumatic brain injury, especially the impact of closed injury on cognitive impairment, as well as the apolipoprotein E ε 4 allele, which is a recognized genetic risk factor for AD and its role in the onset of AD.

Mid term (2010–2016): Key words such as rehabilitation program, plasticity, episodic memory, face name associations, plasticity, and executive function emerged, reflecting a shift in research focus to exploring systematic rehabilitation training programs and focusing on cognitive function, especially memory rehabilitation. At the same time, attention began to be paid to the mechanism of brain plasticity in rehabilitation.

In recent years (2017 present), keywords such as exercise program, virtual reality, quality of life, functional connectivity, and balance have emerged with high intensity, indicating that the application of exercise intervention and brain functional imaging technology in cognitive rehabilitation is increasingly valued. The study of brain functional connectivity has gradually become a new perspective for exploring cognitive function. The systematic review has also shown strong prominence since 2020, indicating that as research continues to deepen, it is necessary to systematically sort out and summarize past evidence.

In recent years, the integration of Virtual Reality (VR) technology with functional neuroimaging, including functional magnetic resonance imaging (fMRI), functional near-infrared spectroscopy (fNIRS), and electroencephalography (EEG), has opened new possibilities for the rehabilitation of Alzheimer’s Disease (AD) patients. By employing fMRI, fNIRS, and EEG during VR training, researchers can monitor real-time brain activity and obtain biomarkers, providing essential data to support personalized rehabilitation plans (Ansado et al., 2020). Additionally, high-precision image-rendered immersive VR training simulates daily scenarios to improve spatial cognition and emotional regulation in AD patients, significantly enhancing patient compliance and rehabilitation outcomes (Manera et al., 2016). Furthermore, EEG-based emotion-adaptive VR technology elevates the therapeutic experience by analyzing emotional states in real-time; the VR system can adjust the virtual environment based on EEG signals to align with the patient’s emotional needs (Seo et al., 2020).

New directions have emerged in quality-of-life interventions for Alzheimer’s disease. Early planning tools, like the Life-Planning in Early Alzheimer’s and Other Dementias (LEAD) Guide, support patients and caregivers in making personalized decisions ahead of cognitive decline, enhancing caregiver confidence and alleviating their burden (Dassel et al., 2023). Mindfulness-based interventions improve emotional self-regulation in patients, aiding them in adapting to cognitive changes in the early stages of Alzheimer’s and thereby enhancing their quality of life (Giulietti et al., 2023). Robot-assisted rehabilitation uses interactive technology to provide cognitive and daily activity support, fostering greater patient independence (Moshayedi et al., 2023). Together, these approaches highlight a diverse trend in Alzheimer’s quality-of-life interventions that integrates personalization, emotional management, and daily support, offering a more comprehensive path forward in rehabilitation.

## 5 Discussion

In this study, we evaluated the hotspots and development patterns of AD rehabilitation research. Between 2000 and 2023, a total of 1,284 articles were retrieved from WoSCC and analyzed using software such as Microsoft Excel (2019 version) and CiteSpace (6.1. R6 and 5.6. R5 versions).

### 5.1 General information

The annual publication volume and trends may provide foresight for the development and progress of research (Ma et al., 2021). Since 2000, research on AD rehabilitation treatment has gone through three stages: rapid growth, platform stability, and another upward trend, indicating that new technologies may help existing research break through bottlenecks and open up new directions for AD rehabilitation research.

From the distribution of countries/regions, the United States occupies a leading position in terms of publication volume, citation frequency, and betweenness centrality in the world; Among the top 10 institutions with the highest publication volume, the University of Toronto has the highest publication volume, but its betweenness centrality is lower than that of the University of California system, Johns Hopkins University, and University of London in the United States, indicating that the United States and the United States play an important decisive role and promote cross institutional collaboration in this research field.

In terms of magazine contributions, NEUROPSYCHOLOGICAL REHABILITATION has published the most articles, but its impact factor and JCR partition are relatively low. Although LANCET, JAMA-JOURNAL OF THE AMERICAN MEDICAL ASSOCIATION, and LANCET have published fewer articles, their IF ranks in the top three. The results of the double graph overlay knowledge graph in journals show that clinical medicine and psychology are the main citation areas for AD rehabilitation research, while psychology, education, sociology, nursing, kinematics, and rehabilitation are also continuously being studied and deepened as citation areas.

As for the author, Clare L. has the highest number of publications and citations, making significant contributions to cognitive rehabilitation, non pharmacological treatment, and cognitive deficits in schizophrenia. However, its mediating neutrality is relatively low, which may be related to its more focused research direction and smaller collaborative network. Representative articles by Clare L include “Non pharmacological therapies in Alzheimer’s disease: a systematic review of efficacy (2010),” which systematically evaluate the impact of non pharmacological treatment on the quality of life of patients with Alzheimer’s disease and related diseases and their caregivers; “Rehabilitation of executive function: An experimental clinical validation of Goal Management Training (2000)” validated the effectiveness of Goal Management Training (GMT) in improving executive function deficits in patients with traumatic brain injury (TBI); A meta-analysis of cognitive deficits in adults with a diagnosis of schizophrenia (2005) analyzed the cognitive deficits in patients with schizophrenia.

Reference clustering analysis suggests that clustering mainly focuses on the diagnosis, evaluation, screening, and intervention of AD, and has strong universality and citation significance. The 10 highly cited references mainly focus on early screening tools, diagnostic criteria and processes, and non pharmacological treatment methods such as neuropsychological rehabilitation for Alzheimer’s disease. However, the analysis of the references highlights that early research on Alzheimer’s disease mainly focuses on cognitive training and rehabilitation efficacy in mild cognitive impairment (MCI) and early AD patients; mid term focus on evidence-based medicine for cognitive rehabilitation training, as well as personalized and specific cognitive rehabilitation plans for functional deficits; recently, guidelines have been used as a new high citation field.

### 5.2 Hotspot frontier analysis

In terms of keyword frequency, “mild cognitive impairment” and “cognitive impairment” indicate that the study distinguishes different stages of AD development and explores them separately; Cranial magnetic stimulation, cognitive rehabilitation, and exercise intervention indicate that non pharmacological treatment methods are becoming research directions for improving AD symptoms.

Keyword clustering analysis suggests that research focuses on the basic disease characteristics of AD, the neurological and psychiatric symptoms and diagnostic criteria of different types of dementia syndromes, intervention and rehabilitation measures for AD, and common issues affecting self-care ability in the elderly population. This provides clues for future research directions. The keyword emergence analysis shows the evolving trend of research hotspots in the field of AD rehabilitation. Early attention should be paid to the impact of traumatic brain injury on cognitive impairment, as well as the role of risk genetic factors such as apolipoprotein (Apo) E ε 4. The mid-term focuses on exploring rehabilitation training programs and the mechanism of brain plasticity in rehabilitation. Recently, attention has been paid to the application of exercise intervention and brain functional imaging techniques represented by brain functional connectivity in cognitive rehabilitation.

## 6 Limitation

This study analyzed research trends in specific fields by reviewing articles in the Web of Science core journal collection of English core journals indexed by SCIE. Although these results provide valuable insights, there are certain limitations. One is the lack of articles published in other databases or languages for research, and in the future, its coverage can be expanded by utilizing databases such as PubMed or Scopus. Secondly, keyword and reference analysis may not provide sufficient information to reveal deeper research motivations and specific research processes. Thirdly, due to the high citation rate of classic articles, newer high-quality literature may find it difficult to rank in the top 10. Fourthly, bibliometric analysis is more suitable for extracting trends at the macro level rather than analyzing at the meso and micro levels.

## 7 Conclusion

Over the past 20 years, a total of 1,284 research papers on AD rehabilitation have been published worldwide, showing an overall trend of increasing year by year, indicating that AD rehabilitation research still has significant research value. Distinguishing non pharmacological treatments at different stages of development is a research hotspot in AD rehabilitation; Sports intervention, brain functional imaging techniques represented by brain functional connectivity, virtual reality, and quality of life are research directions that need attention.

## Data Availability

The original contributions presented in the study are included in the article/supplementary material, further inquiries can be directed to the corresponding author.
